# Photoluminescence Intermittency from Single Quantum Dots to Organic Molecules: Emerging Themes

**DOI:** 10.3390/ijms131012487

**Published:** 2012-09-28

**Authors:** Erin A. Riley, Chelsea M. Hess, Philip J. Reid

**Affiliations:** Department of Chemistry, University of Washington, Box 351700, Seattle, WA 98195, USA; E-Mails: eriley1@uw.edu (E.A.R.); hessc@uw.edu (C.M.H.)

**Keywords:** semiconducting nanocrystals, single molecule spectroscopy, power-law, Auger recombination, blinking, biexciton, multiexciton, fluorescence, proton transfer, electron transfer

## Abstract

Recent experimental and theoretical studies of photoluminescence intermittency (PI) or “blinking” exhibited by single core/shell quantum dots and single organic luminophores are reviewed. For quantum dots, a discussion of early models describing the origin of PI in these materials and recent challenges to these models are presented. For organic luminophores the role of electron transfer, proton transfer and other photophysical processes in PI are discussed. Finally, new experimental and data analysis methods are outlined that promise to be instrumental in future discoveries regarding the origin(s) of PI exhibited by single emitters.

## 1. Introduction and Overview

Since the first optical detections of single molecules pioneered by Moerner [1] and Orrit [2], single molecule (SM) spectroscopy has become a fast growing multifaceted field of research. Technological advances have made SM detection almost routine, and have opened new areas of study including single-particle tracking [3–6], super-resolution spectroscopy [7–9], and SM fluorescence correlation spectroscopy [10–12]. Multi-color excitation techniques [13–15], two-photon excited fluorescence [16], and vibrational spectroscopy through surface enhanced Raman spectroscopy are also rapidly emerging areas of SM spectroscopy [17,18].

Despite the numerous applications of SM techniques, the connection of SM emission to molecular-level phenomena remains a vexing problem in SM spectroscopy. Under continuous photoexcitation the majority of single emitters demonstrate photoluminescence intermittency (PI), also referred to as “blinking”, with the emitter stochastically alternating between emissive (*on*) and non-emissive (*off*) durations. These transitions reflect the population and subsequent decay of anon-emissive or “dark” state. Determining the nature of the dark state and the mechanism(s) for its production and decay requires that a connection between PI and underlying molecular photophysics be established. Typically, this connection is made by analyzing histograms of the durations of *on*- and *off*-event durations [19]. This analysis has shown that for a subset of SMs PI arises from the population and decay of a triplet state. For these systems the *on*- and *off*-duration histograms decay exponentially with time constants for intersystem-crossing and triplet-state decay, respectively [20–22]. PI described by first order rate kinetics represents the minority of SM blinking, most *on*- and *off*- duration histograms instead decay as power-law distributions of the form *t*^−α^ where α is the power-law exponent with values observed in the range 1–2. For some SMs the power-law distribution extends over 5 or more decades in time, consistent with the rate constants for dark state population and decay evolving in time, which is also referred to as distributed kinetics [23]. Since the first report of power-law distributed PI for CdSe/ZnS (core/shell) nanocrystals (quantum dots) [24], identifying the origin of “power-law statistics” has been an ongoing pursuit in SM spectroscopy [25]. In addition to quantum dots (QDs), power-law statistics have been observed for a variety of emitters including fluorescent proteins [26,27], nanorods [28], carbon nanotubes [29], polymer chains [30], and organic dyes [31]. The near-ubiquitous observation of power-law PI has spurred attempts to develop a unified model of this phenomenon [32]. Of the models proposed, charge transfer has emerged as the most promoted mechanism for PI [33]. However, in recent years compelling evidence has emerged showing that distributed kinetics may result from a multitude of mechanisms, and direct challenges to the long-standing charging hypothesis for PI in QDs have appeared in the literature.

In this review we explore the current understanding of SM PI in QDs (with CdSe cores in particular) and organic luminophores. Although this area of SM spectroscopy has been previously reviewed [25,33,34], our focus is on studies from recent years. During this time the discovery of highly suppressed QD emission in so called “giant QDs” has motivated many to take a closer look at the fundamental photophysical processes that give rise to PI. Additionally, giant QDs have made plain the presence of long-lived intermediate intensity states, and subsequent measurements of smaller QDs have shown that many of the same phenomena are resolvable and simply went unnoticed in previous studies. The “charging model” for PI in QDs and current challenges to this model will be presented. For organic luminophores the role of electron transfer, proton transfer, conformational relaxation, and other processes that may contribute to PI will be explored. Two themes have emerged during the past few years of PI studies: a divergence from near-ubiquitous power-law PI statistics, and the development of more robust methods for the collection and statistical analysis of PI data. Exploration of these themes will be a focus of this review.

## 2. Photoluminescence Intermittency in Quantum Dots

### 2.1. Early Work

Current photophysical models describing the PI exhibited by semiconductor nanocrystals or “quantum dots” (QDs) can be traced back to the work of Efros and Rosen. In this work the dark state was assigned to an ionized state of the QD formed by trapping of the photoexcited electron [35]. This idea was extended by Verberk and co-workers to explain the general features of PI in QDs, and in particular, the differences in *on*- and *off*-event duration statistics observed for CdSe QDs with and without a ZnS shell [25,36]. For both materials the *off*-duration histograms best fit to power-law, but the *on*-duration histograms were exponential for CdSe core-only QDs and power-law for CdSe with a ZnS shell (CdSe/ZnS). The model developed to explain this result assigned the dark state to an ionized QD with a hole localized in the QD core and an electron transferred to a nearby acceptor or “trap” site in the surrounding matrix. Although the ionized QD can absorb a photon, Auger decay (fast charge-induced non-radiative recombination of the exciton) renders the QD non-emissive. The QD returns to a neutral emitting state when the trapped electron is transferred back to the QD. The model describing PI in these materials assumed spherical symmetry and a uniform distribution of trapping sites around the QD. In this limit, the trapping probability *P_T_*(*r*) should decrease exponentially with particle-trap distance (*r*):

(1) $$P_{T}(r)=\alpha e^{-\alpha r}$$

Modeling electron transfer as a tunneling process, α corresponds to the decay length associated with the tunneling barrier ( $\sqrt{2m_{e}\Delta V}/\hbar$). Once trapped, the back-tunneling probability also decays exponentially, but with a different decay length, β:

(2) $$P_{BT}(r)=\beta e^{-\beta r}$$

The average time (*T*(*r*)) for decay of the non-emissive state is given by:

(3) $$T(r)=T_{0}e^{-\beta r}$$

Performing a change of variables to determine the probability distribution of recovery times, *P*(*T*), yields:

(4) $$P(T)=\frac{\alpha }{\beta T}{\left(\frac{T_{0}}{T}\right)}^{\frac{\alpha }{\beta }}$$

The above expression predicts that decay of the non-emissive state will be distributed according to an inverse power-law with exponent 1 + α/β. The ratio of α/β is related to the tunneling barriers as:

(5) $$\frac{\alpha }{\beta }=\sqrt{\frac{V_{m}-V_{e}}{V_{m}-V_{T}}}$$

In the above expression *V_m_* is the potential energy of the electron in the matrix, *V_e_* is the potential energy of the electron in the excited state of the QD, and *V_T_* is the potential energy of the electron in the trap. The existence of a trapped state requires that the potential energy of the trap be greater than the potential energy of the electron such that α/β < 1. This relatively simple model provides an explanation for the power-law distribution of *off*-durations (Equation 4), and also predicts that power-law exponents should lie in the range 1–2. Furthermore, the model predicts that the *on*-durations should also decay exponentially since the probability for formation of the charge-separated state is exponentially distributed (Equation 1).

**Figure 1:**
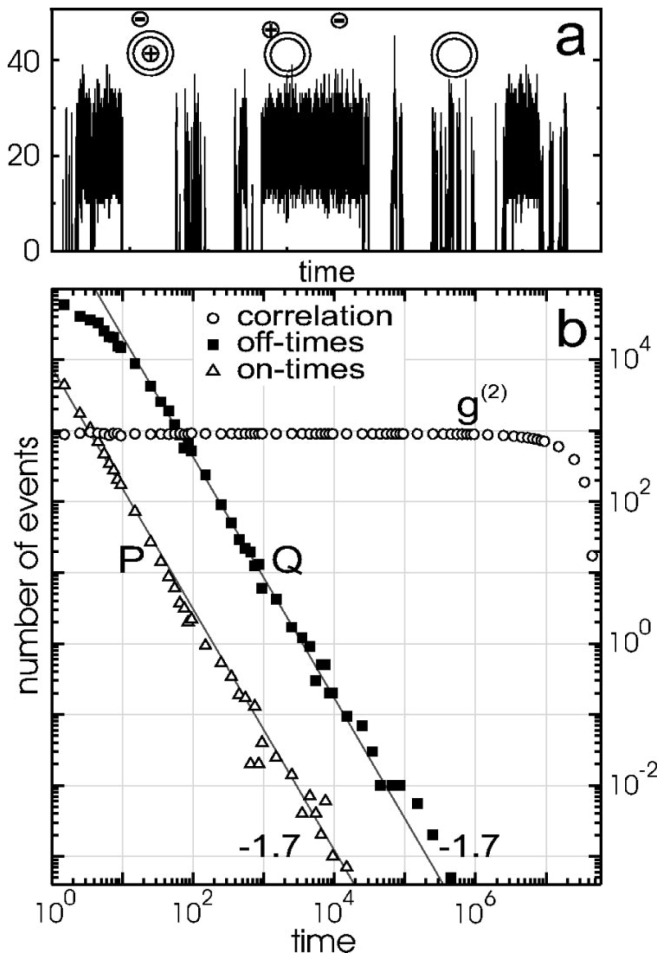
Charging model for quantum dot (QD) blinking from the original paper by Verberk *et al.* [36]. (**a**) Simulated intensity trace for model of capped QDs. Simulations are run for hole trapping probability ɛ = 0.2; (**b**) The *on*- and *off*-event duration distributions are power-laws with exponent −1.7. The distribution of *off*-event durations has been shifted by a factor of 50 s for clarity’s sake. The correlation function appears flat. Reprinted with permission from: Verberk *et al. Phys. Rev. B*, 2002, *66*, 233202. © 2002 by the American Physical Society.

For CdSe/ZnS QDs the authors extended this model to include an additional emitting state to explain the existence of prolonged *on*-durations and the emergence of the power-law distribution. This state is ionized, where the electron is trapped in the matrix just like in the dark state, but energy transfer promotes the hole out of the core to traps states in the shell or at the shell surface such that the QD core remains emissive because the extra carrier cannot promote fast Auger decay. In this case, a Coulomb blockade created by the nearby trapped hole prevents subsequent ionization resulting in prolonged *on*-event durations. Power-law distributed *on*- and *off*-event durations are predicted assuming that in the ionization step the probability the hole being trapped in the shell is ɛ (extended *on*-event), and the probability the hole being trapped in the core is 1–ɛ (*off*-event). The extended *on*-event again ends with back electron transfer where the trapped electron recombines with the hole. The simulated “blinking” trace derived from this model is shown in Figure 1 and power-law statistics are evident for both the *on*- and *off*-event duration distributions by the linear decay of probability on a log-log plot. In addition to providing a working hypothesis for PI exhibited by QDs, this model outlined a host of areas for further investigation:

1. If electron transfer occurs through tunneling, then both the *on*- and *off*-event duration distributions should be temperature invariant.
2. Depending on the location of the hole relative to the electron, multiple emission intensity or “gray” states may be observed.
3. The power-law exponent depends on the potential energy of the electron in the trap site; therefore, blinking statistics should demonstrate dependence on the dielectric constant of the surrounding environment.
4. Shell quality and thickness should impact blinking statistics.
5. The elimination of charge trapping sites should result in an absence of PI.

This “charging model” has been tested by experiments exploring the effect of the QD environment on blinking, and modified PI statistics consistent with the predictions outlined above were observed [33,37–43]. The environmental-dependence of PI is generally explained by hindering ionization through either the passivation or elimination of trapping sites in the dielectric medium, or at the surface of the QD. Some success has been achieved in connecting the power-law exponent to a stabilizing field factor that depends on the bulk dielectric constant [44], and also on the cross-over time for power-laws truncated with exponential tails [45]. Recently, details of how the carriers are released from trapped states have been theoretically investigated [46,47]. Despite mounting experimental evidence in support of the charging model one fundamental aspect of this model has been difficult to prove; namely, that the ionized state is dark because of Auger decay. While environmental effects are an essential aspect of any PI study, especially the long standing concern of the contribution of order-disorder in power-law statistics, this review’s scope is limited to recent discoveries on the nature of the QDs themselves.

Several other models have been advanced that do not rely on long lived trap states. For example, random-walk models have been developed that are capable of describing the power-law distributed PI exhibited by QDs. In these models, diffusion along the reaction coordinate connecting the emissive and dark state results in power-law statistics. A very attractive aspect of these models was the direct connection between the power-law exponent and the dimensionality of the reaction coordinate, *d*, as α = 1 + *d*/2, with the propensity of power-law coefficients assuming a value of 1.5, consistent with a one-dimensional reaction coordinate [25,48]. These models have been extensively developed by Tang and Marcus to include spectral diffusion, and the fall-off from power-law behavior at longer duration times (so-called “bending tails” of *on*- and *off*-event duration distributions) [44,48,49].

### 2.2. Blinking Suppression, Gray States, and Broken Power-Laws in Giant QDs

**Figure 2:**
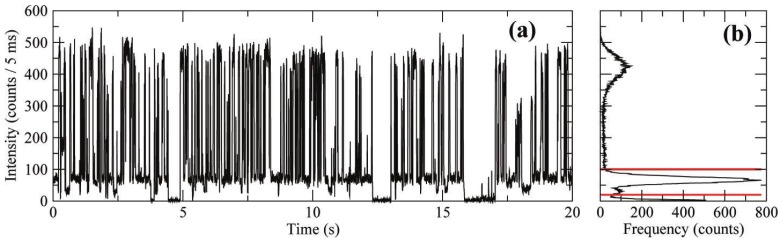
Photoluminescence intermittency trace from a CdSe/CdS nanocrystal (core radius 1.9 nm and 3.5 monolayers of CdS): (**a**) intensity as a function of measurement time; (**b**) histogram of the measured intensity shows two distinct emitting states above the background. Reprinted with permission from: Gomez *et al. ACS Nano*, 2009, *3*, 2281. © 2009 American Chemical Society [52].

Early work on QDs established that the PI exhibited by these materials is characterized by transitions between single emissive and non-emissive states [19,25,35]. More recently, giant CdSe/CdS QDs (nanocrystals with thick shells > 5 nm or >10 monolayers) have been studied and the complex PI exhibited by these materials has gained a substantial amount of attention. These QDs exhibit decreased *off*-event durations, and an increase in the fraction of QDs that do not blink at all [50,51]. In the context of the charging model, these observations are proposed to arise from the elimination of surface trap states. The giant QDs also exhibited clear “gray” or low-emissive states that lie between the highest intensity state and complete extinction (Figure 2) [50,52]. Evidence for multiple emissive states had also been reported for CdSe/ZnS [53], where low-emissive states were found to correspond to lower-energy emission and shorter lifetimes [54]. Although these gray states are short lived in ZnS capped QDs and require statistical rigor to detect, they are prolonged and visually evident in giant CdSe/CdS QDs allowing for detailed investigations of low quantum yield (QY) states and their role in PI.

**Figure 3:**
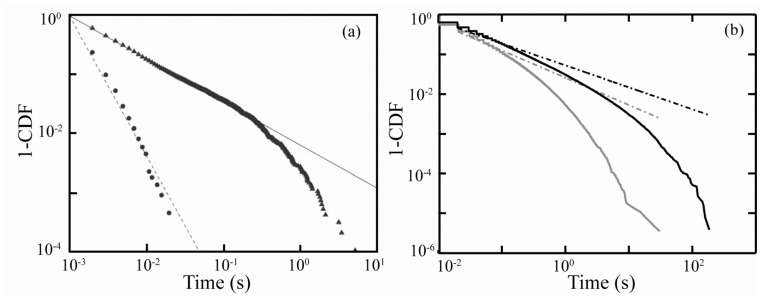
Cumulative distribution function for the *off*-event durations for (**a**) CdSe/CdS nanocrystals with 13 nm diameter core/shell (circles) and CdSe/ZnS (triangles) and corresponding linear fits overlaid [51] (**b**) 4 nm diameter CdSe/CdS quantum dots showing longer durations of *off*-durations (black) and *on*-durations (gray), fits to power-law performed by MLE method [56]. (**a**) Adapted with permission from: B. Mahler *et al. Nature Materials* 2008, *7*, 659. © 2008 Nature Publishing Group.

Recent studies of giant QD PI have revealed a complete departure from power-law blinking statistics. Mahler *et al*. have presented a comparison of the *off*-event durations for giant CdSe/CdS and normal CdS/ZnS QDs. Giant CdSe/CdS QDs demonstrated a linear fall off in the cumulative distribution function on a log-log scale for only one decade in time, and an elimination of long *off*-events [55]. Conventional CdSe/ZnS demonstrated *off*-duration probability over four decades in time, with linearity over the first two decades (Figure 3a) [51]. Our group recently analyzed the blinking of CdSe/CdS QDs with a total size of 4 nm and found deviations from linearity after the first decade in time, inconsistent with power-law statistics (Figure 3b). Together these studies suggest that for CdSe/CdS QDs of any size power-law statistics may not hold, calling into question the universality of power-law for QDs [56]. Further analysis on giant CdSe/CdS QDs with 1.9 nm cores and 3.5 monolayer shells have revealed that single exponential distributions are more appropriate for describing the durations of bright, gray and dark states [52]. Amecke and Cichos have also resolved a dim state (4% QY) in 5 nm diameter CdSe/ZnS QDs with event durations that are exponentially distributed, indicating that the phenomenon had been overlooked in previous experiments [57]. The evolution of *on*-event durations being exponentially distributed for CdSe core-only, to power-law distributed in core/shell QDs, and then returning to exponentially distributed in giant QDs has yet to be accounted for in the literature.

### 2.3. Blinking Suppression and Exciton Lifetimes

**Figure 4:**
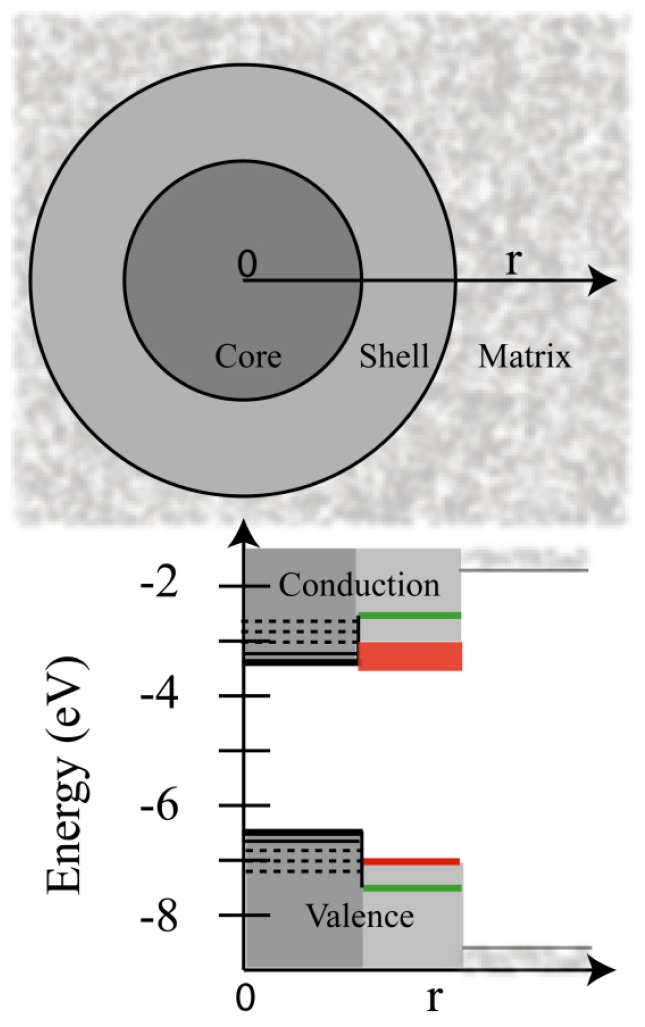
Valence and conduction band alignment for CdSe core quantum dots with ZnS (green) shell and CdS (red) shell. CdSe valence band is located ~−6.8 eV and conduction band at −4.8 eV. CdS is ~−7 eV and ~−4.9 to −5.2 eV (depending on synthesis), and ZnS −7.4 and −3.4 eV. The close match in energy of the conduction bands of CdS and CdSe cause the electron to be delocalized in the shell, as opposed to ZnS where the electron is confined in the core. Matrix potentials (gray) are arbitrarily depicted.

Initial measurements of the lifetimes and photoluminescence QYs of the gray and bright states of giant CdSe/CdS QDs spurred many studies into the role of Auger decay in PI. A central observation to emerge from these studies was that as the CdS shell thickness increases, the lifetime of the bright state also increases from ~20 ns to a limiting value of ~65 ns [50,51]. In addition, the onset of PI suppression was accompanied by the increase in exciton lifetime. The exciton lifetime is dependent on the electronic structures of the core and shell, and is clearly an important component in any description of QD photophysics. The QD shell is typically a semiconductor material with a band gap greater than that of the core which serves to increase the photoluminescence QY. This increase presumably reflects the isolation of the core from the surrounding matrix, limiting photo oxidation, and potentially a reduction in surface trap sites [58]. Similar effects have also been accomplished through the use of various capping ligands [42]. The shell can directly impact the photoluminescence spectrum of the QDs by changing the confinement barriers of the electron and hole wave functions. Figure 4 presents the positions of the valence and conduction bands for CdSe QDs with a ZnS [59] or CdS [60] shell. Photoexcitation of CdSe produces an exciton which is simply an electron in the conduction band and hole in the valence band. If the conduction band of the core is lower in energy than that of the shell (e.g., CdSe/ZnS), then the electron is energetically confined to the QD core. In contrast, when the conduction band of the shell and QD are energetically close (e.g., CdSe/CdS) the electron can delocalize in the shell as evidenced by the band-gap decreasing in energy with increased shell thickness for CdSe/CdS [51]. Another consequence of the increased volume of the QD is that the overlap of the carrier wavefunctions decreases, thereby reducing the exciton recombination rate. This manifests as the increased exciton lifetime (at least twice that of CdSe/ZnS QDs) measured in the giant QDs of CdSe. A comprehensive review on the synthesis and properties of different core/shell architectures for a variety of materials was recently published [58].

### 2.4. Auger Decay in Quantum Dots

#### 2.4.1. Auger Decay and the Gray State

The reduction in exciton recombination rate with increased carrier separation is also relevant to non-radiative decay processes. The relative QY and lifetime of the gray state can be used to estimate the non-radiative (or Auger) decay rate. One prediction of the charging model is that the Auger decay rate must be significantly greater than the radiative decay rate to result in a non-emissive state. In the low-power regime (below exciton saturation) Spinicelli *et al*. have estimated the QY of the CdSe/CdS bright state to be >95%. Relative to the bright state, the gray state has an estimated QY of ~19%, and lifetime of ~8.5 ns [50]. The photoluminescence decay of similar CdSe/CdS QDs was also measured by Gomez *et al*. who reported a smaller average gray-state lifetime of ~5.2 ns [52]. The gray state was interpreted by Gomez *et al*. as well as others to be an ionized QD (a so-called “trion”) with an Auger lifetime of ~10 ns and a radiative lifetime of 45 ns [52,61]. This interpretation was also advanced by Amecke and Cichos for CdSe/ZnS QDs where a 4% QY intensity state was attributed to a stronger confinement potential for the electron [57]. The Auger lifetimes of giant CdSe/Cds [62], giant CdZnSe/ZnS [63] and giant multi-shell [64] QDs are significantly greater than observed in their thin shelled counterparts.

**Figure 5:**
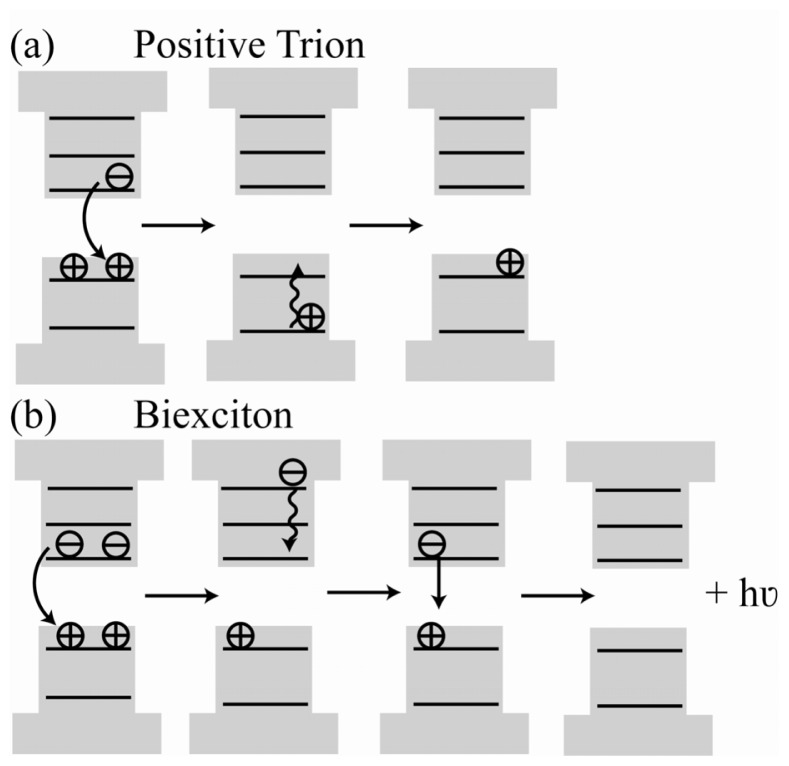
Auger decay mechanism for a positive trion (**a**) and a biexciton (**b**). For the trion, coulomb interaction between the holes promotes one to the conduction band resulting in electron-hole annihilation. The energy is transferred to the remaining carrier to a deep excited state within the valence band (or into a continuum). Fast (a few picoseconds) relaxation back to the valence surface states returns the QD to a charged ground state. Biexciton relaxation is dominated by Auger decay pathways due to the presence of many carriers. One pathway is depicted for biexciton decay, here shown to transfer the excess energy to an electron instead. Fast relaxation of the electron to the lowest conduction states produces a single exciton. The exciton preferentially relaxes radiatively to generate the neutral ground state and the emission of a photon.

Considering a positively charged trion, an Auger process occurs due to the Coulomb interaction between two holes of opposite spin that occupy ground states in the valence band. As a result of this interaction, one of the holes is transferred to the conduction band where electron-hole annihilation occurs, and the energy from this process is transferred to the remaining hole. Consequently, the hole is promoted to an excited state within the valence band. This carrier relaxes quickly (a few picoseconds) back to the surface valence state [65]. An illustration of this is provided in Figure 5a. Auger decay can also occur for biexcitons and multiexcitons corresponding to the simultaneous existence of 2 or more excitons. The Auger decay rate for these states is greater than that of the trion due to the presence of more carriers that can participate in energy transfer. An example of one possible Auger decay pathway for biexciton relaxation is illustrated in Figure 5b. This figure shows the transfer of energy from one exciton pair to the remaining electron, promoting it to a higher conduction band state. The energy is dissipated in a fast relaxation back to the conduction band edge, thereby generating an exciton. The exciton can then radiatively decay to the neutral ground state.

**Figure 6:**
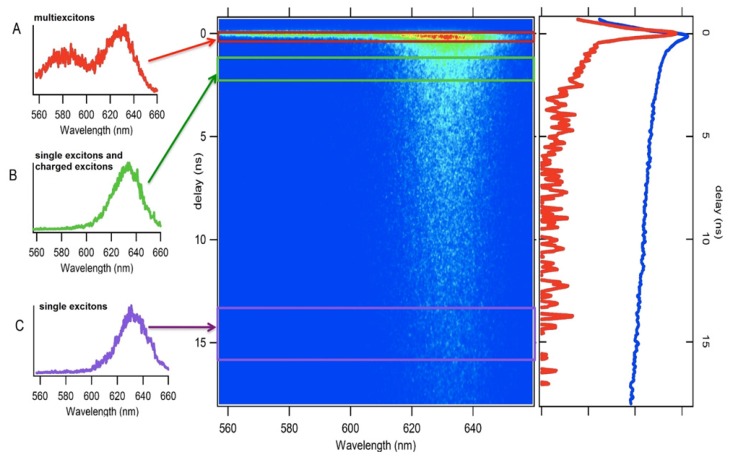
Spectrogram of time resolved photoluminescence from CdSe/CdS nanocrystals 3.6 nm core, 8–10 nm total size [69]. 20 ns collection window, with 90 ps resolution for a laser fluence of 5 mJ cm^−2^. Excitation wavelength 500 nm. Left side panel; photoluminescence spectra as a function of time delay with respect to the laser pulse. (**A**) delay 0 ns, integrated in a 50 ps gate; (**B**) delay 1 ns, gate 1 ns; (**C**) delay 13 ns, gate 3 ns. Right side panel: time decay of the photoluminescence signal measured at 630 nm. (blue trace corresponding to exciton and biexciton emission) and 580 nm (red trace corresponding to multiexciton emission). Reprinted with permission from: M. Marceddu *et al. Nanotechnology* 2012, *23*, 015201. © IOP Publishing Limited.

The degree of reduced Auger decay in QDs exhibiting suppressed PI has been explored through measurements of multiexciton (MX) decay. Due to dominant Auger decay pathways discussed above, the emission from biexcitons (2X) and triexcitons (3X) is characterized by fast photoluminescence decay and low QYs, rendering detection of this emission difficult. However, in giant CdSe/CdS nanocrystals, 2X, 3X and higher order MX lifetimes all increase by almost two orders of magnitude relative to thin shelled QDs indicative of significant suppression of Auger decay [62,66,67]. The emission from 3X is at higher-energy relative to exciton (1X) or 2X emission due to occupation of a higher energy level in the conduction band [68]. This blue shifted feature has been observed in CdSe/CdS QDs at room temperature [67,69] and 4 K [66], as well as in room temperature CdSe/CdZnS QDs [68]. The emission from 3X can be isolated temporally and spectrally as shown in Figure 6 [69]. This figure shows the spectrally resolved photoluminescence decay curve of an ensemble of CdSe/CdS QDs illustrating the fast decay of the high energy 3X feature, followed by the slower decay of the 2X and 1X states, which are energetically similar, but not identical due to exciton-exciton interactions. Emission from charged 1Xs (trions) is potentially convolved with the 1X decay [69]. Emission from 1X and 2X states can be resolved at low temperature where emission of 2Xisshifted by ~10 meV to lower [62] or higher energy [66] relative to 1X when the exciton-exicton interaction [70] is attractive or repulsive, respectively [71]. Emission from2X can also be identified through intensity dependence of the emission decay and the presence of photon bunching. At low temperature, evidence for charged biexciton emission has also been found [66]. Reports on biexciton QYs ranging between 0.1–1 in elongated CdSe/ZnS nanocrystals at low temperature have been reported, where QY heterogeneity is attributed to the shape distribution of the QDs under investigation [72]. The shape of the QD determines the fine structure and, subsequently, Auger rates at low temperatures. The effect of shape and volume effects on radiative lifetimes [73] and biexciton lifetimes [74] has also been investigated for dot/rod structures of CdSe/CdS. Marceddu *et al*. did not find evidence for reduced Auger decay rates for giant CdSe/CdS QDs [69]. However, the exciton lifetime of the QDs used in this experiment was 18 ns, which is shorter than that observed for the onset of blinking suppression, and inconsistent with reduced Auger decay generally observed in giant CdSe/CdS QDs [75].

#### 2.4.2. Alloying and Volume Effects

Cragg *et al*. have recently investigated the role of the core/shell interface in Auger decay, and their simulations predict that gradual grading of the confining potential can decrease the Auger decay rate by 3 orders of magnitude relative to structures with abrupt, square well boundaries [65]. Grading can be achieved by alloying at the core/shell interface to minimize the mismatch in lattice size. The softer boundaries dampen the high frequency components of the hole ground-state wavefunctions thereby decreasing the Auger transition probability. Softening of the potential can be accomplished by rounding the shape of the potential through alloying and by increasing the size of the core. Therefore, the core/shell interface is an important factor in determining Auger decay rates [76].

**Figure 7:**
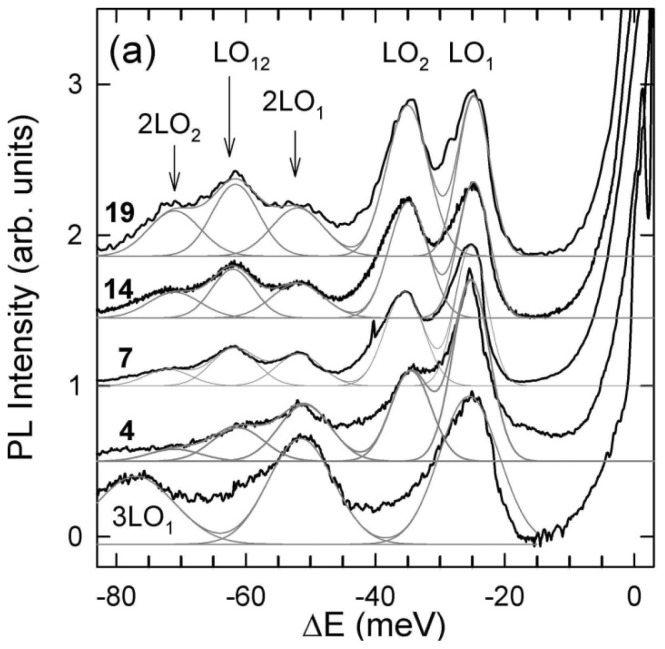
Fluorescence line narrowing spectrum of CdSe/CdS nanocrystals [77]. Core only (bottom curve) and four samples with core radius 1.5 nm and different shell thicknesses 1.6, 2.8, 5.6, 7.6 nm and corresponding values in mono-layers are reported next to each curve. Gray lines show Gaussian fits to the FLN spectra that account for contributions of phonon replicas associated with the LO modes of CdSe (LO_1_), CdS (LO_2_), and CdSeS (LO_12_). Reprinted with permission from: García-Santamaría *et al. Nano Lett.*, 2011, *11*, 687. © 2011 American Chemical Society.

Garcia-Santamaria *et al*. have explored the effect of shell thickness and alloying on Auger decay rates using fluorescence line narrowing [77]. A summary of their study is provided in Figure 7. The presence of an alloy can be identified in the fluorescence line-narrowing (FLN) spectrum through phonon-assisted transitions involving longitudinal optical (LO) modes. Each signal consists of a primary peak for the LO assisted exciton emission, and Stokes shifted phonon overtones. The phonon modes are material specific such that CdSe and CdS modes can be identified, as well as combinations of the two indicating the presence of an alloy. Figure 7 presents the FLN spectra as a function of shell thicknesses for 1.5 nm CdSe cores. The core only spectra show primary LO_1_ assisted emission at −0.25 eV relative to excitation, with two overtones 2LO_1_ (−0.50 eV) and 3LO_1_ (−0.75 eV). As the shell is added, LO_2_ modes from the shell appear at −0.35 eV and overtone 2LO_2_ (−70 eV). The presence of an alloy is evidenced by the combination of the LO_1_ and LO_2_ modes at −60 eV. This mode is assigned to a “graded” region where the mismatch between the CdSe and CdS lattice is reduced. Formation of an alloyed layer results in a 2 orders of magnitude increase in the biexciton Auger lifetime, τ_2A_ (from ~250 ps to ~31 ns). As the shell becomes thicker, the LO_12_ mode becomes more pronounced, indicating consumption of the core material in the alloy layer. The alloy layer increases in thickness until ~9 monolayers are achieved, at which point the Auger lifetime makes much smaller gains: a 3 fold increase in τ_2A_ from 31 ns at 9 monolayers to 90 ns at 14 monolayers. This increase was found to directly correspond to a three-fold reduction in the electron-hole overlap integral [77].

Further evidence connecting reduced Auger decay rates to PI was recently reported by Ghosh *et al*. Here, studies of CdSe/CdS revealed that the onset of blinking suppression occurs with fewer shell layers for larger cores, and that the smallest cores require larger shells to achieve suppression [75]. This is consistent with theoretical predictions that a wider hole confinement potential should reduce Auger decay [65]. PI suppression was only observed for CdSe/CdS core/shell QDs with lifetimes (~65 ns) that are longer in comparison to their core-only or thin shelled counterparts [75]. Similar results were obtained by Vela *et al*. who observed large changes in the power-law exponent for both the *on*- and *off*-duration distributions with a transition in shell size from thin shells of< 5 monolayers (α ≈ 1.5), to intermediate shells of 6–12 monolayers (2 < α < 2.5), to large shells of >13 monolayers (α ≈ 3) [78]. The authors compared CdSe/CdS QDs to those with additional shell layers of CdZnS/ZnS and found that these additional layers did not alter the PI statistics. In CdSe/CdS nanorods, increased volume alone was found to decrease the quantum yield, independent of core size, indicating QD shape is an important aspect in determining decay pathways [79].

### 2.5. Challenges to the Charging Model for QD PI

**Figure 8:**
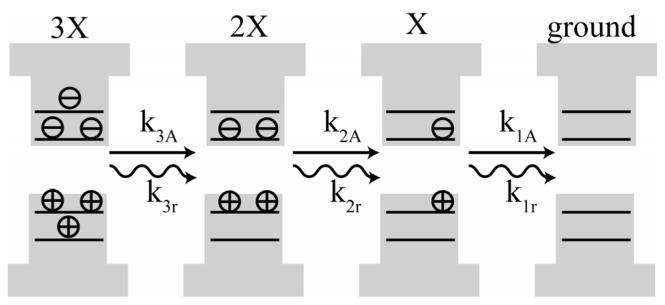
Cascading decay of a triexciton (3X). Multiexciton stepwise decay can occur radiatively with rate constants *k_ir_*, or by Auger decay with rate constant *k_iA_*. Multiple photon detections per excitation pulse result from the decay of multiexcitons, the overall radiative decay lifetime is a convolution of the exciton and multiexciton kinetics.

A significant challenge to the foundations of the charging model is the demonstration that Auger decay alone is insufficient to create a dark state for a singly charged QD. Building from earlier work on the isolation of the MX emission in CdS/CdZnS QDs [80], Zhao *et al*. directly measured the QYs and radiative lifetimes of the X, 2X, 3X, and dark states [68]. The results of these measurements were then compared to the predictions of the charging model. Figure 8 illustrates the relaxation of 3X states occurs through sequential population of the 2XandX states. Each state can decay radiatively, or non-radiatively by Auger decay. The relative radiative decay rate for 3X is greater than that 2X or X; however, the increase in Auger decay rates dictates that the lifetimes of 3X and 2X are very short.

The relative quantum yields of the 2X to X states and trion states (X^+/−^) can be estimated by determining the transition probabilities for various decay pathways that are available to these species [68]. The exciton recombination rate is determined by the overlap of the electron (*ψ_e_*) and hole (*ψ_h_*) wavefunctions. The electron and hole wavefunctions can be treated separately in nanocrystals in the strong confinement regime, or when the confinement energy of the electron and hole is much larger than the Coulombic interaction between them. The electron and hole wavefunctions qualitatively resemble particle in a sphere wavefunctions. Generalizing to the cubic structure, the valence bands of CdSe QDs arise from *p*-atomic orbitals, which are inherently six fold degenerate when spin is included. Spin orbit coupling results in separation into a four-fold degenerate level with total unit cell angular momentum (*J* = *l* + *s*) of *J* = 3/2, and a deeper band level that is two-fold degenerate with *J* = 1/2. This results in four primary valence bands that have approximately equal occupation probabilities corresponding to *J_m_* = 3/2, 1/2, −1/2, −3/2. The total angular momentum, *F*, of the QD hole states is the sum of the angular momentum quantum number (*L_h_* = 0, 1, 2; corresponding to S, P, D orbitals) and *J_m_* such that *F* = *L_h_* + *J_m_*. Unlike the valence band, the conduction band is comprised of many states that can be populated, and the electron is treated as a free particle. Because of this, the valence structure is important in determining the recombination pathways. The decay of X is proportional to the square of the transition dipole |〈*ψ_h_*|*μ⃑*|*ψ_e_*〉|^2^. The relative radiative rate constants can be estimated using the Wigner Eckart theorem, |〈*F_h_,J_h_*|*μ↔*|*F_e_,J_e_*〉|^2^, where subscripts denote the quantum numbers for the hole (*h*) and electron (*e*). For the four primary valence bands described above, the relative strengths of the transition dipole moments for X decay are given by the nonzero matrix elements [68]:

(6a) $${\left|\langle 3/2,3/2\left|\mu_{1}^{\left(1\right)}\right|1/2,1/2\rangle \right|}^{2}=k_{r}$$

(6b) $${\left|\langle 3/2,1/2\left|\mu_{0}^{\left(1\right)}\right|1/2,1/2\rangle \right|}^{2}=\frac{2}{3}k_{r}$$

(6c) $${\left|\langle 3/2,-1/2\left|\mu_{-1}^{\left(1\right)}\right|1/2,1/2\rangle \right|}^{2}=\frac{1}{3}k_{r}$$

where *k_r_* is a constant. The *F* = 3/2, *J_m_* = −3/2 valence band has a transition dipole moment of zero. The average over all four states gives an approximate radiative rate for X of *k_x_* = *k*_r_/2. For a positive trion (X^+^), two *J_m_* states are occupied (one for each hole) resulting in 6 possible valence band configurations, each of these configurations has two possible recombination pathways with the electron (one for each hole) which sum for the overall rate constant for that configuration. Using the equations above for each contribution from individual electron-hole pairs, an average of *k_x_*_+_ = *k_r_* is obtained. For a negative trion (X^−^), there are four valence configurations, just like neutral X, but two possible interactions per band due to the electron spins (1/2 and −1/2). This also results in *k_x_*_−_ = *k_r_*. For 2X, 6 valence band configurations are possible as for X^+^, but each hole can recombine with either electron, resulting in four recombination pathways per configuration to yield *k_2x_* = 2*k_r_*.

Radiative recombination is the preferred decay pathway for X such that *k_x_* ≫ *k_A_*, where *k_A_* is the rate constant for Auger decay. In the charging model the decay of 2X and X^+^/X^−^ is dominated by *k_A_* ≫ *k_r_*, where *k_A_* is assumed to be approximately the same for 2X and X^+^/X^−^. Using these assumptions, the relative emission QYs of 2X and X^+^/X^−^ emission can be found and compared experimentally. In summary, the prediction of the charging model is that the QY of X^+^/X^−^ should be greater than half of 2X.

**Figure 9:**
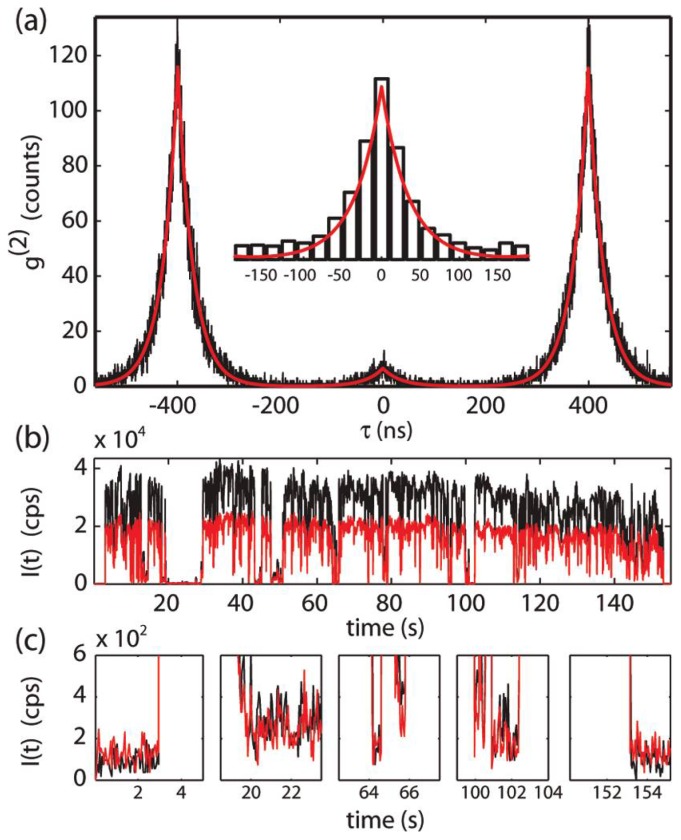
(**a**) Measured, unnormalized *g*^(2)^ from a single CdSe/CdZnS NC under 12 μJ/cm^2^ pulsed excitation, representative of the *N* ≪ 1 limit [81]. The normalized integrated area of the center feature, *g*_0_^(2)^ = 0.06, is a direct measure of the 2X to X quantum yield ratio of this QD. Inset is a 20 ns binned detail of the center peak. Red lines are a fit to the sum of three two-sided exponentials; (**b**) Time traces of intensity in start and stop channels (black and red) during the g^(2)^ acquisition; (**c**) Details showing *off*-event intensities. The events in the first and last panel correspond to switching the excitation laser on and off. Reprinted with permission from: Nair *et al. Nano Lett.*, 2011, *11*, 1136. ©2011 American Chemical Society.

Recently the 2X QY was measured by fitting the photon cross correlation histogram *g*^(2)^ generated from PI collected in a time correlated single photon counting set up (TCSPC) under low laser fluencies (Figure 9) [81]. In this experiment, single-particle emission is delivered to a beam splitter and collected by two detectors (referred to as a Hanbury Brown-Twiss set-up). The cross correlation histogram is generated by determining the time delay between photon arrivals on the two detectors. The time delay between detectors can be used to detect multiple emissions per excitation cycle. The common assumption is that in the low-power limit a single QD will not have intensity at zero time delay, *g*_0_^(2)^, because emission arises from a single exciton. The presence of resolvable intensity at *g*_0_^(2)^ at low powers is commonly attributed to the presence of a second nearby emitter, and taken as evidence that more than one particle is present in the illuminated volume. Surprisingly, this study demonstrates that this assignment is likely in error, and that modest intensities for *g*_0_^(2)^ are consistent with 2X emission. Since the decay of 2X to the ground state is stepwise (Figure 8), radiative decay for each step results in potentially two photons emitted for each excitation event, and therefore intensity at *g*_0_^(2)^. The relative 2X QY is determined from the integrated area of the *g*_0_^(2)^ peak in the normalized *g*^(2)^ histogram collected in the low power regime [81]. A similar approach can be taken to measure the 3X emission through additional spectral decomposition of the emission [68]. The relative dark-state QY can also be found directly from the ratio of dark state to emissive state intensities.

Applying the above methods to CdSe/CdZnS QDs, Zhao *et al*. measured a 2X QY of ~0.12 relative to X, and a relative dark-state QY of 0.01, much less than the predicted value of >0.06. These results stand in direct opposition to the predictions of the charging model. Additional analysis of the 3X dark state found an intensity ratio of charged 3X+/− to neutral 3X to be ~0.1, much lower than the predicted value of >0.25 demonstrating that the dark state non-radiative decay rate is faster than can be explained by traditional Auger process involving just one additional carrier [68]. Nair *et al*. has found a wide range of heterogeneity in the 2X emission QY for a variety of samples [81]. Park *et al*. have also found a large distribution of 2X QYs in giant CdSe/CdS QDs using the same method as Nair *et al*. with values ranging from <0.05 to ~0.9 for QDs that do not blink [67]. These large variations in the 2X emission QY are consistent with substantial variation in Auger decay rate constants, which has been assumed to be directly related to blinking suppression; however, these results call into question the validity of this assumption. Careful analysis of higher excitonic states in QDs is an important area of further studies involving the origin of PI in QDs.

Closer inspection of the low QY dark state also indicates that the charging model is of limited utility in explaining its photophysics. Zhao *et al*. noted a distribution in dark-state QYs between different QDs in the same sample [68], and similar heterogeneity has been observed in the fluorescence trajectories of single QDs. Cordones *et al*. directly measured the dark-state lifetime in CdSe/ZnS QDs and found lifetimes of ~4 ns for *off*-event durations lasting >600 ms [82]. The authors also note that the lifetimes actually increase with the length of *off*-durations within a single QD. This is consistent with changes in the non-radiative rate constants in the *off*-state, which is not expected if Auger decay solely determines the non-radiative decay rates of dark states. A similar result for *off*-event lifetimes was observed by Rosen *et al*. in CdSe/CdS/ZnS QDs where an absence of QD size dependence on the *off*-event lifetimes was also observed [83]. Spectroelectrochemistry has recently been used by several groups to directly interrogate the effects of injected charges to determine the trion radiative rates for CdSe [84,85] and CdSe/ZnS [85] QDs. Results show that charged states may have QYs as high as 15%, too high to account for a dark state. Galland *et al*. used spectroelectrochemistry to demonstrate that PI can arise from charging and discharging of the QD core, where low QY states possess short lifetimes; and also PI with QY changes that are not accompanied by significant lifetime changes [86]. The authors interpret this to arise from charge fluctuations in the electron-accepting surface sites. The authors also found that PI could be suppressed by the application of an appropriately high electric field. Spectroelectrochemistry was recently used to investigate the lifetimes of alloyed CdSeS/ZnS QDs, where the lifetimes were found to become longer and more distributed with increased potential [87]. Other investigations into the dark state recovery process in CdSe/ZnS QDs have found that the dark state decays through light activated pathways and dark pathways. Through incorporating large dwell times between intervals of continuous excitation, Baker *et al*. show that dark state recovery in the absence of illumination occurs much more slowly than under continuous illumination [88]. This is consistent with the findings of Osborne *et al*. who observed that ensembles of CdSe/ZnS QDs demonstrate photoluminescence activation when under continuous illumination followed by a combination of photo deactivation and activation [89]. The light assisted and dark pathways for dark state recovery are another avenue that will require further investigation.

### 2.6. New Mechanisms

#### 2.6.1. Multiple Charging Mechanism

In the charging model, the dark state was attributed to ionization to form a trion where emission is completely quenched through Auger decay. The presence of gray states in giant QDs complicates this assignment by requiring the introduction of another long lived state, which has so far has also been assigned to a trion with a reduced Auger decay rate [52,57]. To explain the dark state, Gomez *et. al.* proposed a multiple charging mechanism mediated by the ionized state, where further charging mediated by surface traps drives rapid Auger decay [52]. Multiple charges could account for the relationship of 3X, 2X and dark state QYs observed by Zhao *et al*. where the traditional Auger mechanism was not applicable [68]. Califano recently tested this proposal theoretically, and found that multiple charges resolves the experimental discrepancy of the dark state and biexciton QYs [90]. The generation of multiple charges should be sensitive to the excitation energy and intensity.

#### 2.6.2. Multiple Recombination Centers

Another proposed mechanism for QD PI is activation and deactivation of trapping states at the surface or in the core/shell interface of the QD. Frantsuzov *et al*. recently modeled the interaction of the exciton with an environment of two level systems (TLS) [91]. Surface states that lie just above the valence band can be occupied by the hole if thermal energy is transferred to the exciton in a phonon assisted absorption, thereby promoting excess energy to both carriers. Relaxation of the electron back to the lowest conduction band state transfers more energy to the hole promoting it to higher surface states [92]. Subsequent recombination of the two carriers occurs non-radiatively. Unlike the long-lived carrier trapping invoked in the charging model, this mechanism involves a non-radiative decay pathway for exciton recombination with the hole trapped briefly. The authors propose that there are about 10 quenching states that can be activated or deactivated to alter the number of available non-radiative decay pathways. This activation/deactivation of quenching states is predominately due to light induced changes in the interface atoms of the QD that changes the interaction between the nearby TLS. The total non-radiative decay rate is then determined by a transition matrix containing the interaction of each TLS with the exciton, and the number of non-radiative decay pathways that are available. This model accounts for the evolution in dark state lifetimes, and other features not discussed in this review such as memory [93], spectral diffusion [94], bending tails, and threshold dependence on the cross-over time and power-law exponents. This mechanism also has an appeal of generality for the broader problem of understanding SM intermittency for other chromophores, and perhaps even broader in terms of the study of intermittent processes in complex systems in their own right.

## 3. Photoluminescence Intermittency in Organic Luminophores

In addition to QDs, the PI exhibited by organic luminophores has also been extensively studied. As mentioned in the introduction, early studies of PI in organic materials involved systems for which the triplet state served as the dark state [95–99]. The role of the triplet state was confirmed by emissive and non-emissive event durations that were exponentially distributed in accord with the known rates of triplet state production and decay. However, organic luminophores for which the triplet state is the dark state represent the minority, with the majority of organic emitters demonstrating non-exponential distributions of *on*- and *off*-event durations. Similar to the QDs described above, the majority of luminophores exhibit event duration distributions that appear to follow a power law. The observation of power-law distributed event durations is taken as evidence that the rate constants for dark-state production and decay evolve over the observation time, a condition commonly referred to as “dispersed kinetics”. Much work over the past decade has sought to identify the processes responsible for dispersed kinetics. A recent review of this subject focused on charge transfer mechanisms which have been widely invoked as the origin of PI in a large variety of organic luminophores [33]. In addition to charge transfer, in this review we present experiments and conclusions that suggest alternative mechanisms for PI including conformational relaxation, proton transfer, and spectral diffusion. Other environmental effects on emitter photophysics will also be discussed. The central theme to emerge from these studies is that the relationship between the guest and host is perhaps the most important factor in determining the origin of PI.

### 3.1. Electron Transfer

Given the proposed charging model for QD PI described earlier, it is not surprising that electron transfer has emerged as the leading hypothesis for PI in organic materials. In the electron-transfer model the dark state is assigned to a radical form of the luminophore resulting from electron-transfer with the surroundings [33]. As long as the molecule exists as a radical it is non-emissive, with back-electron transfer resulting in reformation of the emissive neutral form of the luminophore. The first study to fully explore the electron-transfer hypothesis involved rhodamine 6G (R6G) dissolved in poly(vinyl alcohol) (PVOH) [100]. In this study, triplet states were identified as possible intermediary states promoting charge transfer between R6G and the PVOH host. Changes in R6G emissivity with excitation intensity between air and nitrogen suggested that emissivity changes must reflect the formation of an additional dark state, proposed to be the radical ion form of R6G. Radical formation was confirmed by the observation of a photoinduced spin-1/2 species observed in ESR experiments on heavily dyed films. The distribution of electron-trapping sites in PVOH was proposed to provide for a distribution of forward and back electron transfer rates that manifest as power-law blinking statistics. The proposed triplet intermediate hypothesis and power-law distribution was revisited in PI studies of Atto565 (another rhodamine derivative) on glass under air and nitrogen environments where power law distributed event durations were also observed [101]. The connection of power-law distributed *on*-and *off*-event durations to triplet states that promote charge separation has been explored by others [102]. Recently, the role of triplet states in promoting charge-separated dark states was explored in perylenediimide (PDI) and terrylenediimide (TDI) dispersed in poly (methyl methacrylate) (PMMA). Here power-law distributed emissive and non-emissive event durations were observed with excitation at 520 nm, yet no blinking was observed with lower-energy 647 nm excitation. This result was attributed to secondary photoexcitation of the chromophore in the lower-energy triplet state with 520 nm excitation resulting in population of a higher-energy triplet state from which electron transfer occurs [103]. Direct photo initiated electron transfer from the luminophore to the surroundings without the participation of the triplet state has also been proposed to result in PI [101]. Despite the number of studies on organic luminophore PI that invoke radical formation, direct evidence for radical production is widely lacking.

A clear test of the electron-transfer model is to change the redox properties of the host system such that electron-transfer becomes thermodynamically prohibitive. This idea was explored by Clifford *et al.* for the organic dye Atto647N dissolved in three polymers: Zeonex, poly(vinyl carbazole) (PVK) and PVOH [104]. Analysis of the *on*- and *off*-event durations in PVK and Zeonex demonstrated substantial differences in the power-law exponent consistent with the redox properties of the host influencing PI. Recent studies by Yasuda *et al.* on *N*,*N*′-Dipropyl-1,6,7,12-tetrakis (4-*tert*-butylphenoxy)-3,4,9,10-perylenetetra-carboxydiimide (BP-DPI) incorporated into droplets of *n*-octane observed an absence of PI that was attributed to rapid electron-hole recombination observed for alkanes, consistent with minimal stabilization of charge in this solvent [105]. Additionally, studies of Atto655 incorporated into modified DNA chains with hole trapping sites demonstrated that increasing the distance between Atto655 and the trap site provided for an increase in the fluorescence correlation time consistent with charge transfer impacting PI [106]. In recent studies of cyanine chromophores in the presence and absence of oxygen, the dark-state lifetime increased with increasing reduction potential of the luminophore [107]. Finally, radical anion formation was attributed to cause both intermediate and dark states depending upon the solvation of anion by the surrounding matrix for calix[4]arene-linked perylenebisimide dimers resulting in possibly three different kinds of blinking determined by the local environment [108]. In total, these studies are consistent with electron transfer between the luminophore and environment resulting in PI.

### 3.2. Proton Transfer

Proton transfer has not been a widely invoked hypothesis for organic luminophore PI since this process was considered to occur on a timescale significantly shorter than that typically explored in PI experiments. Our group has been investigating the role of proton transfer in the PI exhibited by rhodamine-type chromophores isolated in single crystals of potassium acid phthalate (KAP) [102,109–113]. Comparative studies of violamine R (VR) emission in PVOH and KAP found that the temperature dependence of VR PI was markedly different in these two environments. Specifically, in PVOH a very modest temperature dependence of the power-law exponents describing emissive and non-emissive event durations was observed [114]. In contrast, for VR in KAP both the *on* and *off*-event durations increased with increased temperature. Given that earlier studies had identified electron-transfer as the origin of PI for rhodamine chromophores in PVOH, the different temperature dependence in KAP suggested that something other than electron transfer must be responsible for PI. Proton transfer was developed as an alternative to electron-transfer, with this hypothesis largely motivated by the hydrogen bonding structure of KAP and the incorporation of VR into KAP through substitution into this hydrogen-bonding network [115].

**Figure 10:**
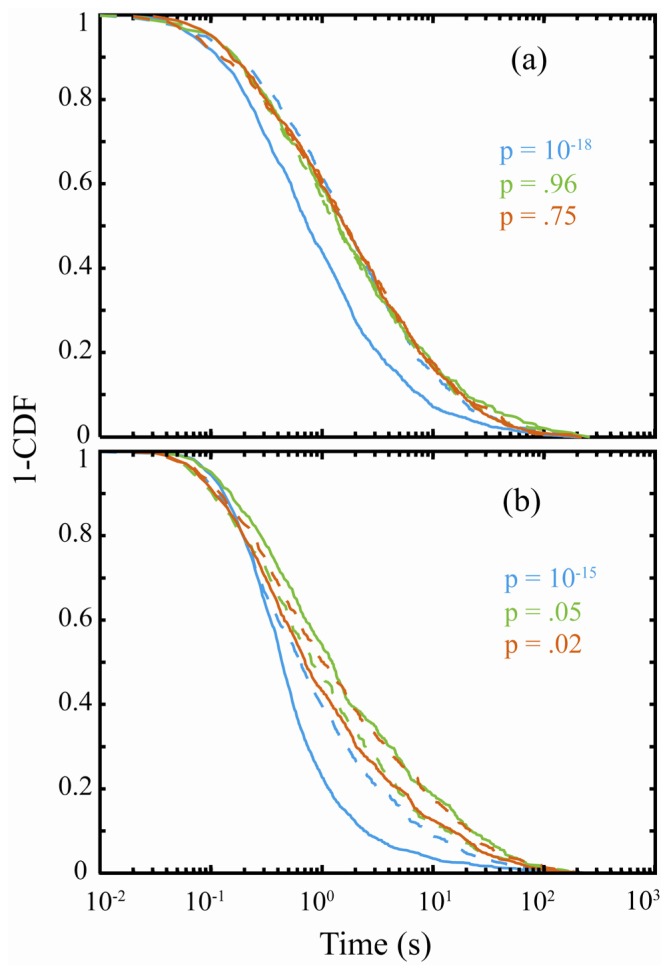
Complimentary CDF’s of *on*-(**a**) and *off*-(**b**) event durations for VR in KAP and DKAP at all three temperatures [116]. Dashed lines are DKAP and solid lines are KAP at room temperature (blue), 45 °C (green), and 60 °C (red). The *p*-values correspond to the comparison of the complimentary CDFs for DKAP and KAP at the same temperature. *p*-values > 0.05 indicate that there is a greater than 5% probability that the data arise from the same underlying probability distribution. Adapted with permission from: Riley *et al. J. Phys. Chem. B*, 2012, doi:10.1021/jp306392e © 2011 American Chemical Society.

**Figure 11:**
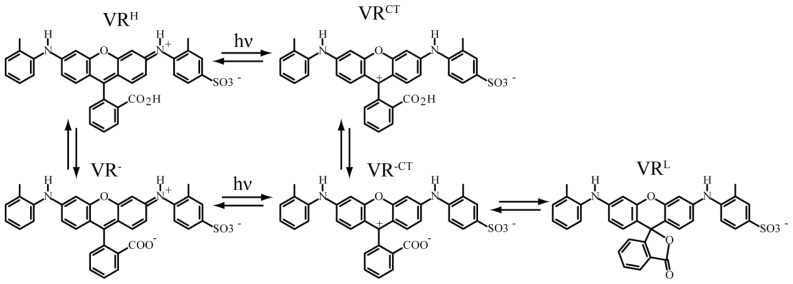
Protonation states of ground state violamine R and photoinduced intramolecular charge transfer states responsible for the non-radiative decay pathway of the dye [116]. The colorless Leuco form is accessed through the charge transfer state, and is a possible dark state for VR molecules overgrown in potassium acid phthalate crystals.

The role of proton transfer in the PI exhibited by VR in KAP was recently demonstrated in isotopic substitution experiments on VR isolated in protonated and deuterated KAP (DKAP). In this study, the PI exhibited by VR in KAP and DKAP was analyzed using new statistical methods [56] to determine if the underling probability distribution functions describing the *on*- and *off*-durations were modified with isotopic substitution. This analysis involved the statistical comparison of complimentary cumulative distribution functions (CDFs) derived from the PI data obtained for VR in KAP and DKAP at three temperatures (23 °C, 45 °C, and 60 °C) [116]. The results of this analysis are presented in Figure 10. Figure 10a shows a comparison of the *on*-interval complimentary CDFs for VR in KAP and DKAP. The likelihood that the two data sets are derived from the same probability distribution function is quantified by the *p*-value, where a *p*-value > 0.05 means that the data have a greater than 5% probability of being derived from the same PDF. The analysis presented here demonstrates that the *on*-duration data for VR in KAP and DKAP are statistically different at room temperature (*p*-value of 10^−18^), but become statistically equivalent at elevated temperature (*p*-values > 0.75). In contrast, the *off*-duration data (Figure 10b) for VR in KAP versus DKAP are statistically different at all temperatures. The statistically significant difference in VR PI in KAP and DKAP establishes that proton-transfer results in PI. The proposed proton-transfer model for VR PI involves two emissive protonation states and a colorless ring-closed or leuco form of the chromophore. Transitions between these forms (Figure 11) are responsible for the variation in emissive and non-emissive periods observed in PI studies. Finally, the PI temperature dependence was attributed to an increase in the distance between the donor and acceptor accompanying expansion of the crystal.

**Figure 12:**
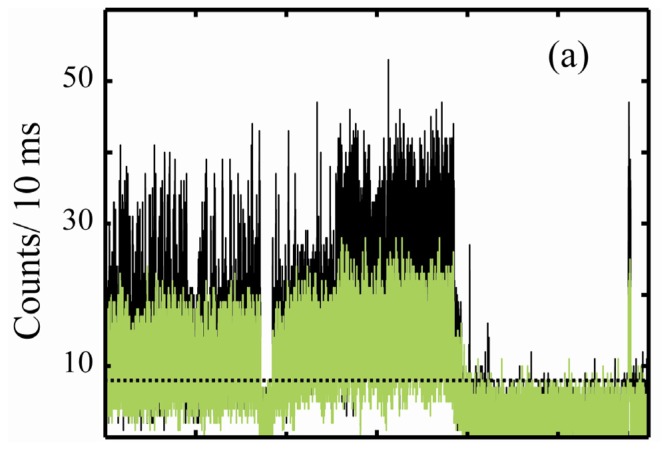

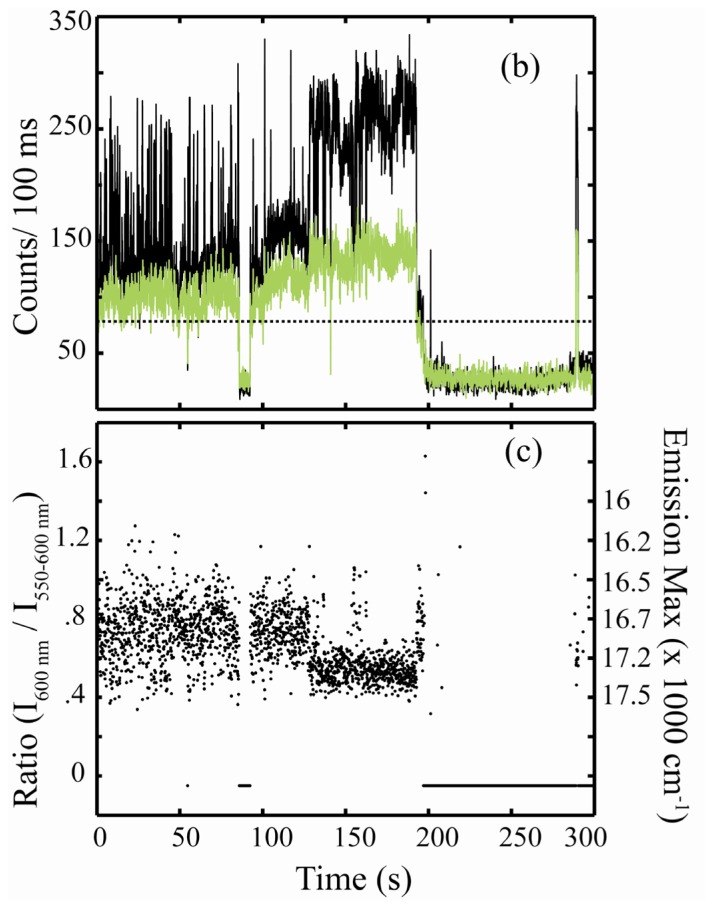
Emission trace of VR collected on two detectors with different spectral windows of 550–600 nm (black) and >600 nm (green) [116]. (**a**) Trace collected with10 ms bin time; (**b**) Same trace re-binned to 100 ms; (**c**) Ratio of the two channels in (**b**), and estimated emission maxima corresponding to ratio values. VR^H^ (see figure X) emits at ~16,200 cm^−1^ and VR^−^ at ~17,200 cm^−1^. Evolution in the ratio of the two channels is correlated with intensity changes; this is consistent with a change in protonation state of the dye corresponding to the reduced absorbance cross section of VR^H^ relative to VR^−^. Correlation of intensity between the two channels with change in ratio also agrees with this assignment. Anti-correlation of the black and green channels is evidence for spectral diffusion. Reprinted with permission from: Riley *et al. J. Phys. Chem. B*, 2012, doi:10.1021/jp306392e © 2011 American Chemical Society.

In addition to these studies, we have recently found that an interchange between the two emissive protonation states is also distributed in time, and can be directly observed in the emission of the SMs. This was determined by splitting the single molecule emission spectrally between two detectors one with a window of 550 nm–600 nm and the other > 600 nm. The deprotonated form of the dye, VR^−^ (Figure 11) emits at ~17,200 cm^−1^, and the protonated form, VR^H^, emits at ~16,200. Furthermore VR^−^ has approximately twice the absorbance cross section of VR^H^. Proton transfer manifests as a large increase in counts on the 550 nm–600 nm detector, and a smaller correlated increase in the >600 nm detector (Figure 12a,b). The overall ratio indicates conversion between the two species spectrally (Figure 12c), supported by the correlated intensity jumps expected from the change in absorbance cross section. Spectral diffusion of a single protonation state would manifest as anti-correlation between the two channels. Investigating the intermittent exchange between two emissive forms is a rational pathway to understanding single molecule photophysics without needing to identify a dark state. If exchange between two emissive forms also demonstrates dispersed kinetics, then the functional form of the probability distributions for switching could lend insight into the inherent nature of intermittent processes.

### 3.3. Spectral Diffusion and Environmental Effects

Spectral diffusion was one of the first photophysical phenomena observed from SMs [20]. In many PI studies, a variety of emissive intensities are observed rather than a single “bright” state of fixed intensity and a single non-emissive state. The variation in emissive intensity has been assigned to spectral diffusion which results in a change in the absorption cross section of the luminophore at the excitation wavelength employed and a corresponding change in emissive intensity. Since spectral diffusion of single molecules appears as a change in emissive intensity, it is an important component in any discussion of PI [112]. In our studies of VR in KAP we observed multiple emissive intensities in blinking traces consistent with a distribution of dielectric environments within a crystal host [112]. The relationship between spectral diffusion and fluorescence intensity was also recently investigated by Krause *et al.* for perylenediimide (PDI) in dipyridylperylenediimide (DPP). The energy difference between spectra before and after a diffusive transition were measured, and it was shown that single molecules can cover more than one-third of the overall energy landscape available in a single diffusive event [117].

Spectral diffusion arises from fluctuations in the surrounding environment, or from reorientation of the molecule relative to the environment, such that the electronic properties of the luminophore are altered. The connection between the environment surrounding the luminophore and PI has been recently explored for a variety of systems. The influence of a polymer’s free volume on PI has recently been explored by Masuo *et al.* through studies of charge transfer complexes [118]. Monocarbazolyl and dicarbazolyl donor compounds interacting with a 1,2,4,5-tetracyanobenzene acceptor in various polymers were studied, and histograms of lifetime differences between two consecutive measurements were analyzed. This analysis demonstrated that the extent of lifetime variation was directly related to the free volume of the polymer. This study concluded that the dark-state corresponds to temporary dissociation of the charge transfer complex which can occur only when sufficient free volume is available. In contrast, studies by Suzuki *et al.* on DiI (1,1′-dioctadecyl-3,3,3′3′-tetramethylindocarbocyanine) in different polymers found that the measured lifetimes for this system do not depend on polymer [119]. A similar observation was made by Yoo *et al.* in studies of π-stacked perylenediimide (PDI). Measured lifetimes of PDI were different for dimers and trimers dissolved in poly (butyl methacrylate) (PBMA) and PMMA polymers. Since PBMA has a large nonpolar side group that hinders chain packing, more free volume is available in this polymer allowing for changes in dimer orientation [120]. Other examples of environmental effects include butadiyene linked porphyrin dimers incorporated into PMMA of different percent weights [121]. Single step photobleaching statistics were enhanced in lower-density PMMA, but blinking was less frequent. Kruger *et al*. found that lower environmental pH reduces the PI of light harvesting complexes accompanied by an increase in of the number of intermediate intensity states populated [122].

### 3.4. Conformational Relaxation

In contrast to QDs, organic molecules introduce the added complexity of conformational relaxation which can contribute to PI. In particular, conformational relaxation is extremely important when describing the photophysics of fluorescence polymers, and an excellent overview of this area has been reported by Vacha and co-workers [123]. With regards to molecular luminophores, early studies by Moerner and co-workers on fluorescent proteins observed that for GFP (green fluorescent protein) blinking arose from different conformational states formed by isomerization [124]. This finding was in agreement with other work that concluded that cis/trans photoisomerization could play a role in photophysics of GFP [125,126]. Recently, two butenolide and two pyrrolinone derivatives were found to undergo a cis-trans photoisomerization associated with their “photoswitching” behavior [127]. However, in comparison to electron and proton transfer, only a limited number of systems have shown a direct connection between PI and conformational relaxation.

## 4. Conclusions and Outlook

Recent studies of SM PI in both QDs and organic luminophores have promoted a shift away from old paradigms for PI, namely the near universality of power-laws in blinking statistics and the proposal that a single mechanism can explain the PI exhibited by all SMs. In QDs these advances have been made through improved synthesis and analysis methods, allowing for a detailed connection between composition and photophysical properties. This has led to a greater understanding of the charged emitting states of QDs and as well as the connection of Auger decay rates and PI, which remains an open topic of exploration. Through tailoring the size, shape, and shell thickness many a more comprehensive picture of QD photophysics and PI is beginning to emerge. However, many questions remain unanswered. What causes the evolution from exponential *on*- and *off*- duration distributions (as well as gray states) to power-law like distributions? Is power-law as ubiquitous as it first appeared to be, or are there better distributions to describe the data? Reduced Auger decay rates are correlated with suppressed PI, but are they the cause of this suppression? What metric should be used to distinguish an apparent “gray state” from the multitude of statistically possible emissive states? To what degree are datasets biased by rejecting QD data with greater multiexciton QYs or more dominant gray states? Is there correlation between bright, dark and gray states, or is population and decay independent? What is the difference in mechanism for light assisted dark state decay from spontaneous dark state decay? We would also note that in this review we have covered only the most popular nanocrystal materials, which raises the obvious question as to what extent with the PI exhibited by QDs with CdSe cores can be compared with QDs based on other semiconducting materials. Once again, careful investigations of nanocrystal composition and the corresponding photophysical properties of the QD will provide the greatest advances in our understanding of the PI exhibited by these systems.

In recent years the photophysics of organic SMs and their role in the PI exhibited by these materials have attracted more attention. Free from many of the composition ambiguities that hinder studies of QDs, organic SMs remain the most flexible materials in which to systematically investigate the factors that impact PI. In addition, organic SMs exhibit many of the same characteristic features of PI that QDs do, making them complimentary in understanding the phenomenon of intermittency at large. It is doubtful that a catholic mechanism for organic luminophore PI will be established. Instead, an emerging theme is that the origin of PI will depend on the photophysical processes that are available to a specific chromophore, and on the environment surrounding the chromophore. For example, electron transfer may result in PI in one environment, but changing the surroundings such that electron transfer becomes thermodynamically unfavorable could make plain the contribution of other processes to PI. While identification of the dark state will remain a focus of PI studies on organic SMs, other photophysical processes such as spectral diffusion can contribute to intermittency without the formation of a dark state. Understanding the chromophore-environmental interactions that promote spectral diffusion will continue to be an active area of study. Finally, new analysis methods that allow for a statistical comparison of PI data have created the opportunity for researchers to perform quantitative investigations of structural and environmental perturbations on PI. Quantification of the change in PI statistics with a specific perturbation should provide for a more robust test of theories designed to explain the PI exhibited by organic materials.
